# Degradation kinetics of medium chain length Polyhydroxyalkanoate degrading enzyme: a quartz crystal microbalance study

**DOI:** 10.3389/fbioe.2023.1303267

**Published:** 2023-12-14

**Authors:** Fabien Millan, Nils Hanik

**Affiliations:** Institute of Life Technologies, School of Engineering, University of Applied Science and Arts Western Switzerland, Sion, Switzerland

**Keywords:** polyhydroxyalkanoates, depolymerase enzymes, quartz crystal microbalance, degradation kinetics, biodegradable polymers, enzymatic degradation

## Abstract

This study investigates the enzymatic degradation processes of different classes of polyhydroxyalkanoates (PHAs), a group of biopolymers naturally synthesized by various microorganisms. Medium chain length PHAs (mcl-PHAs) are distinguished biopolymers due to their biodegradability and diverse material properties. Using quartz crystal microbalance measurements as a valuable tool for accurate real-time monitoring of the enzymatic degradation process, the research provides detailed kinetic data, describing the interaction between enzymes and substrates during the enzymatic degradation process. Thin films of poly-3-hydroxybutyrate (PHB) and polyhydroxyoctanoate copolymer (PHO), containing molar fractions of about 84% 3-hydroxyoctanoate and 16% 3-hydroxyhexanoate, were exposed to scl-depolymerases from *Pseudomonas lemoignei* LMG 2207 and recombinant mcl-depolymerase produced in *Escherichia coli* DH5α harboring the plasmid pMAD8, respectively. Analyses based on a heterogeneous kinetic model for the polymer degradation indicated a six-fold stronger adsorption equilibrium constant of mcl-depolymerase to PHO. Conversely, the degradation rate constant was approximately twice as high for scl-depolymerases acting on PHB. Finally, the study highlights the differences in enzyme-substrate interactions and degradation mechanisms between the investigated scl- and mcl-PHAs.

## Introduction

Polyhydroxyalkanoates (PHAs) are aliphatic polyesters naturally produced by several microorganisms, including *Bacillus*, *Pseudomonas*, *Azotobacter*, *Hydrogenomonas*, and *Chromatium*. These microorganisms synthesize PHAs intracellularly for carbon and energy storage, typically through the fermentation of carbon-rich substrates. The accumulation process often occurs under unbalanced growth conditions such as carbon excess and limitation of essential elements like phosphorus, nitrogen, or oxygen (Raza et al., 2018). Based on the number of carbon atoms in their monomers, PHAs can be grouped into subclasses. Short-chain length PHA (scl-PHA) consists of monomers with three to five carbon atoms, while medium-chain length PHA (mcl-PHA) contains monomers with 6–14 carbon atoms.

A distinctive characteristic of PHAs lies in the vast range of physicochemical, mechanical, and thermal properties they can exhibit, owing to the broad substrate specificity of PHA polymerases and the myriad of potential monomers (Matsumoto et al., 2009; Amstutz et al., 2019). This versatility has placed PHAs as the largest group among natural polyesters (Kim et al., 2007). The advent of genetically engineered strains and innovations in carbon sources and feeding strategies further enhances our ability to produce tailored PHAs with a more recent focus on the production of mcl-PHAs and their industrial applications (Tortajada et al., 2013; Oliveira et al., 2020; Muthuraj et al., 2021; Reddy et al., 2022). Consequently, these polymers find applications in diverse fields, from bioplastics and biomaterials to medical implants and biofuels (Hazer and Steinbüchel, 2007; Brigham and Sinskey, 2012; Panchal et al., 2013; Li et al., 2016).

The proliferation of petroleum-based plastics in the environment has raised pressing concerns due to their prolonged persistence (Singh and Sharma, 2016). Both terrestrial and marine ecosystems face the mounting challenge of plastic debris accumulation, with marine regions being especially burdened by sizable aggregations known as “plastic gyres” (Derraik, 2002; Law, 2017; Drzyzga and Prieto, 2019). The widespread occurrence of microplastics, tiny fragments resulting from larger plastic breakdown or direct release, intensifies the issue, affecting a great number of species across diverse habitats (de Souza Machado et al., 2018; Hanik et al., 2019; Soo et al., 2021). Current recycling and waste management efforts address only a portion of the total plastic waste, leading to increased calls for sustainable alternatives. In this context, biobased polymers like PHAs are gaining attention. PHAs are naturally produced by microorganisms and, crucially, are biodegradable. Their ability to serve as a direct replacement for many petroleum-based plastic applications while offering a reduced environmental footprint makes them a promising candidate in the quest for sustainable materials. Although PHAs available on the market are more expensive than those derived from petroleum, they have the advantage of not necessarily being derived from nonrenewable energy and fossil raw materials. Their production is not only possible from edible substrates such as sugars or vegetable oils but also substrates obtained from agricultural and food industry waste (Khardenavis et al., 2007). From a financial and environmental point of view, a considerable potential for optimization exists for the manufacturing processes, mainly with the use of bioenergy, biocatalyst, and carbon source from waste (Chen, 2009; Chen et al., 2020). The integration of PHAs into industrial and consumer applications could not only reduce the inflow of non-degradable plastics but also provide a model for how bioengineering can offer tangible solutions to pressing global environmental issues.

However, there are still many economic and technological challenges to overcome in the field of PHAs (Wang et al., 2014). One of them is to develop unique and tailored material properties for specific applications. For example, the degradability of biopolymers is an essential property which needs to be well adapted for biomedical or packaging applications (Gumel et al., 2013; Keskin et al., 2017; Kalia et al., 2021). Biopolymers can undergo degradation through different mechanisms, which depend on the polymer structure and exposure conditions. Physical and chemical degradation are the two main types of degradation. Physical degradation results from physical changes, such as thermal embrittlement and environmental stress cracking. Chemical degradation occurs through chemical reactions, with common examples including photo-induced, thermal, thermal-oxidative, solvolytic, hydrolytic, and biological degradation. Understanding the mechanisms of biodegradation is essential for designing more sustainable and environmentally friendly materials (Meereboer et al., 2020; Silva et al., 2023). In this regard, the monitoring of the biopolymers degradation has become a key area of research.

Secretion of PHA depolymerases by microorganisms represents one of the possibilities for the biodegradation of extracellular PHA in the environment. While many extracellular scl-PHA depolymerases have been purified and well characterized (Handrick et al., 2001; Braaz et al., 2003; García-Hidalgo et al., 2012), only few mcl-PHA depolymerases have been described so far (Schirmer and Jendrossek, 1994; Gangoiti et al., 2012; Martínez et al., 2015). These enzymes play a crucial role in the biodegradation process, as they catalyze the breakdown of the larger polymer molecules into smaller units that can be metabolized by microorganisms. Several techniques have been developed for monitoring the enzymatic degradation of plastics, such as turbidimetric powder and film assay (Timmins et al., 1997a; Timmins et al., 1997b), Taylor dispersion analysis (Chamieh et al., 2015), and Blue-ray-based micromechanical characterization (Ceccacci et al., 2017). However, these techniques often suffer from time-consuming sample preparation and complex experimental set up. Some require the polymer to be formulated into stable suspensions or free-standing films which limits their application to specific mechanical polymer properties for monitoring the enzymatic degradation. One tool that has been proven to be useful for this purpose is the quartz crystal microbalance (QCM). Based on the inverse piezoelectric effect the QCM can be used to measure very small changes in the mass of a material in real time (Sabot and Krause, 2002; Marx, 2003). QCM sensors are commonly 5 or 10 MHz AT-cut quartz crystals with gold electrodes on both sides that vibrate in the thickness-shear mode during measurements, where the two surfaces move in an antiparallel fashion. The use of these sensors as microbalances is based on the linear relationship between the resonance frequency variation and the mass variation of the quartz crystal, which can be described by the Sauerbrey equation (Eq. 1), where Δ𝑓 and 𝑓_0_ represent the normalized frequency variation and the resonant frequency of the fundamental mode, respectively. The active area, the density, and the shear modulus describe the crystal used for the measurement and are represented by *A*, *ρ*_*q*_, and *µ*_*q*_, respectively. The corresponding mass variation is represented by *Δm* (Sauerbrey, 1959; Johannsmann, 2015).

$$\Delta f=-\frac{2f_{0}^{2}}{A\sqrt{\rho_{q}\mu_{q}}}\;\Delta m$$ (1)

Applying this equation to a quartz crystal with a fundamental resonance frequency (RF) of about 5 MHz, assigns a change in the RF of 1 Hz to a mass variation of about 17 ng on the sensor surface. This information is useful for designing experiments that use QCM to monitor the degradation of biodegradable plastics, such as PHA. QCMs have been used to study the enzymatic degradation of polyester films in several scientific publications. Yamashita et al., used the combination of QCM with atomic force microscopy (AFM) to study interactions between an enzyme and a biopolymer film at the molecular level Yamashita et al. (2005). In our experimental set up, a continuously circulating enzyme solution is introduced into a fluidic cell and brought in contact with a PHA coated sensors. The changes in the RF due to the degradation of PHA are monitored over time.

Various models have been developed to characterize the depolymerization kinetics of enzymes, taking into account different mechanisms, kinetics, and environmental factors. The heterogeneity of the reaction (i.e., the interaction between a soluble enzyme and an insoluble substrate in the case of PHA films) requires more complex models. The observed degradation behavior was found to be inconsistent with classical Michaelis-Menten homogenous enzymatic kinetic where both enzyme and substrate are considered as soluble. At high enzyme concentrations, the reaction rate becomes inversely proportional to the enzyme concentration. The heterogenous kinetic model proposed by Mukai et al., assumes that the depolymerase first binds to the substrate surface and then hydrolyzes polymer chains. However, if the adsorption process is faster than the rate-limiting hydrolysis, surface crowding limits the unhindered access of the enzymes catalytic domain to the substrate. This eventually leads to a decrease in the degradation rate of the poly-3-hydroxybutyrate (PHB) film when increasing the enzyme concentration (Mukai et al., 1993). This kinetic model successfully described the experimental data reported for the enzymatic degradation of PHB films by several PHB depolymerases. However, to the best of our knowledge, this approach was never applied to other classes of PHA polymers and their corresponding depolymerases.

In this study, we apply the QCM technique to model the degradation kinetics of PHA films from different PHA classes. The model we use was adapted from Mukai et al. and can be written in the form of the rate equation (Eq. 2), where υ_0_ represents the highest degradation rate observed for the corresponding enzyme concentration [*E*]_*0*_. The adsorption equilibrium constant and the degradation rate constant are represented by *K* et *k'*, respectively.

$$v_{0}=\frac{k^{\prime }K{\left[E\right]}_{0}}{{\left(1+K{\left[E\right]}_{0}\right)}^{2}}\;$$ (2)

We characterize mcl-PHA degradation and compare it with scl-PHA degradation by their respective depolymerases. As far as we know, this is the first detailed study of the enzymatic degradation of mcl-PHA in direct comparison of scl-PHA degradation. In this approach the QCM technique is especially useful as the mcl-PHAs are commonly highly amorphous polymers which are not readily available as free-standing films or stable polymer suspensions (Jendrossek and Handrick, 2002). Based on our experiments, we find a distinguished difference between the scl- and mcl-PHA depolymerases investigated. The results highlight the importance of understanding not only the molecular structure of the enzyme and its substrates but also their degradation mechanism. The applied analytical method allows to distinguish the parameters for adsorption equilibrium and degradation rate, providing a deeper understanding of the key features of the PHA degradation by their specific depolymerases.

## Materials and methods

PHB powder was purchased from Biomer (Germany) and polyhydroxyoctanoate copolymer (PHO), containing molar fractions of about 84% 3-hydroxyoctanoate and 16% 3-hydroxyhexanoate as determined by gas chromatography, was kindly provided by Prof. Dr. Manfred Zinn (University of Applied Science and Arts Western Switzerland). All other chemicals and reagents were purchased from Sigma-Aldrich (Switzerland) and used without any further purification.

### Strains, plasmid, media, and cultivation conditions

*Pseudomonas lemoignei* LMG 2207 was kindly provided by Prof. Dr. Dieter Jendrossek (Stuttgart University, Germany) and received on filter paper blots. The filter paper blots were stored at 4°C. The strain was used for all cultivations in the following sterilized minimal medium (adapted from Stinson and Merrick, 1974): Disodium succinate (15 mM), KH_2_PO_4_ (33 mM), Na_2_HPO_4_ × 2 H_2_O (33 mM), NH_4_Cl (18 mM), MgSO_4_ × 7 H_2_O (2 mM), FeCl_3_ × 6 H_2_O (0.037 mM) and CaCl_2_ × 2 H_2_O (0.045 mM).

*Escherichia coli* DH5α [*supE*44, Δ*lac*U169 (Ø80lacZΔM15), *hsdR*17, *recA*1, *endA*1, *gyrA*96, *thi*-1, *relA*1] was used as host for the expression of modified mcl-depolymerase from *Pseudomonas fluorescens* GK13 with the previously described plasmid pMAD8 (Ihssen et al., 2009). The preculture and agar slant was performed in LB-Amp medium: 5 g L^−1^ yeast extract, 10 g L^−1^ tryptone and 5 g L^−1^ NaCl, 100 mg L^−1^ ampicillin. The bioreactor cultivation was conducted in Terrific Broth (TB): 24 g L^−1^ yeast extract, 12 g L^−1^ tryptone, 4 mL L^−1^ glycerol, 0.25 mL L^−1^ PPG 2000, 0.017 M KH_2_PO_4_ and 0.072 M K_2_HPO_4_, 100 mg L^−1^ ampicillin.

### Colony selection for producers of scl-depolymerases

In order to identify colonies of *P. lemoignei* producing extracellular scl-depolymerases, colonies were incubated on the solid media supplemented with 1.5% w/v of PHB powder at 30°C for 72 h. The selected colonies, distinguished by a circular halo around them, were used to inoculate precultures.

### Preculture preparation

*Pseudomonas lemoignei* LMG 2207 was cultivated in shake flasks for precultures. For the preparation of the minimal medium, CaCl_2_ × 2 H_2_O, FeCl_3_ × 6 H_2_O, NH_4_Cl, and MgSO_4_ × 7 H_2_O were autoclaved separately and mixed at room temperature to avoid the formation of precipitates. The resulting solution was supplemented with 5 g L^−1^ of disodium succinate. A gas-liquid ratio of 5 was respected for all cultivations to keep sufficient microbial culture aeration. Colonies from solid media plates were selected for medium inoculation. These cultures were incubated at 30°C and 160 RPM in an orbital shaker incubator. The biomass growth was monitored by measuring the optical density at 600 nm (OD_600_) of 1 mL samples. The incubation was stopped once the desired OD_600_ was reached.

In a similar way, *E. coli* DH5α strain harboring the plasmid pMAD8 was streaked on LB-Amp Agar at 37°C and incubated overnight, providing single colonies to inoculate LB-Amp medium. The precultures were cultivated overnight at 37°C with 200 RPM agitation.

### Batch cultivation with pH shift

For the optimization of scl-depolymerases production from *P. lemoignei* in a 3 L benchtop bioreactor (KLF, Bioengineering), the cultivation was carried out as reported by Terpe et al. (1999). A preculture of 200 mL with an OD600 of 1.0 was used to inoculate the main culture medium to obtain an OD600 of approximately 0.1. The fermentation was separated into biomass accumulation and depolymerase production phases, differentiated only by the pH setting of 6.3 and 7.6, respectively. The pH adjustment was realized by adding smaller volumes of NaOH 5 M or H_3_PO_4_ 5 M. Furthermore, the process parameters were set to a temperature of 30°C, a stirring speed of 400 RPM, and an aeration of 2 vvm with air. The pO_2_ was prevented to fall below 30% by addition of O_2_ to the inlet gas. Samples of 5 mL were taken regularly during the whole cultivation. The OD_600_ of samples was measured to follow the biomass growth and to apply the pH shift during the late exponential phase. Moreover, the enzyme activity was measured on crude culture medium samples with a spectrophotometric enzyme assay.

For the expression of mcl-depolymerase by *E. coli* harboring the plasmid pMAD8, a stock solution of 0.17 M KH_2_PO_4_ and 0.72 M K_2_HPO_4_ was separately autoclaved and added to the main solution together with the ampicillin. Cultivation conditions in the 3 L benchtop bioreactor were set to 37°C, a pH of 7.0 (adjusted with NaOH 5 M and H_3_PO_4_ 2 M), an aeration of 1 vvm, and agitation starting at 700 RPM and gradually increasing to 1,200 RPM to prevent the pO_2_ to drop below 30%. The bioreactor was inoculated with 100 mL of the LB-Amp preculture. The OD_600_ was monitored and once it reached 6, the temperature was reduced to 25°C and 0.5 mM IPTG was added for induction. After 16 h of induction, cells were harvested and isolated by centrifugation.

### Spectrophotometric enzyme activity assay

To follow the production of extracellular depolymerases, the enzyme activity was monitored using the model substrate *para*-nitrophenyl butyrate (pNPB) according to the spectrophotometric assay described by Rios et al. (2019).

### Isolation and concentration of depolymerases

For scl-depolymerases from *P. lemoignei*, the isolation of extracellular enzymes was performed by centrifugation of the crude culture medium at 2500 × g for 45 min at 4°C. The enzymes present in the supernatant fraction were concentrated by two consecutive ultrafiltration (UF) cycles using centrifugal concentrator tubes of 15 mL with a 50 kDa molecular weight cut-off regenerated cellulose membrane (Vivaspin Turbo 15 RC 50kDa, Sartorius). The concentrator tubes were filled with 10 mL of supernatant and centrifugated 5000 × g for 15 min at 4°C for the first concentration step and 7 min at 4°C for the second step.

For mcl-depolymerases from recombinant *E. coli*, the crude culture medium was centrifuged at 7000 × g for 30 min at 4°C. The pellet was resuspended in a lysis buffer consisting of 30 mM Tris-HCl (pH 8.0) and 1 mM EDTA. The cells were disrupted using a French press at 1,000 bar for two cycles, keeping the mixture on ice throughout the process. The mixture was then centrifuged at 9384 × g for 15 min at 4°C to obtain the supernatant containing the depolymerase. The solution was concentrated using a 30 kDa centrifugal concentrator tubes (Amicon, Millipore). The final step of purification was performed by preparative chromatography (Äkta start, Cytiva) using a high-flow amylose resin (n°E8022S from neb, Bioconcept Ltd.).

### Protein quantification

For protein quantification, a sample volume of 1 µL was directly deposited onto the measurement pedestal of a Nanodrop spectrophotometer (DS-11 FX +, DeNovix) and measured at 280 nm. As the exact composition of the mixture of depolymerases was not known, the concentrations were calculated based on the percent extinction coefficient of bovine serum albumin provided by the device manufacturer (0.667 L g^−1^ cm^−1^).

### PHA thin film preparation

PHA solutions were prepared for the coating step of thin films on quartz crystal sensors. Surface electron microscopy (SEM) was employed to support the development of a suitable coating procedure (Supplementary Figure S1). The solution was made from PHB powder or PHO film solubilized in trichloromethane (1% w/v) and heated at 50°C for 1 h to fully solubilize the polymer. A fraction of 10% v/v of toluene was added to the solutions to optimize the evaporation speed and film thickness during the spin coating process. For dynamic spin coating of quartz crystal sensors, the speed of the spin coater (Ossila Ltd.) was set at 4,000 RPM and 140 µL of polymer solution was cast on the rotating sensor center. The spinning was continued for 40 s, followed by drying the coated sensor in a vacuum oven at 37.5°C and 50 mbar for 20 min. Finally, the film was examined visually for any imperfections such as irregularities, pinholes, or scratches.

### QCM measurements procedure

Experiments were carried out with a QCM (Q-1, openQCM) allowing the measurement of the RF, dissipation, and sensor temperature. The sensor type was a 5 MHz quartz crystal with a blank diameter of 13.95 mm, a frontal gold electrode diameter of 11.20 mm, an AT-fundamental cut, a quartz density of 2649.7 kg m^−3^, and one gold electrode on both sides. For data acquisition and real-time monitoring, the openQCM Q-1 Python Software (GUI Python version 2.0) was used.

### Film stability assessment

Before any degradation experiment, the film stability was assessed by monitoring the RF variations of coated quartz sensors dispensed in circulating buffer solution. Chemical-resistant tubes (Tygon 2375-C, Saint Gobain) of 1.6 mm inner diameter, connected the measuring module to a magnetically stirred reservoir. Phosphate buffer at pH 7 was introduced into the reservoir and a constant flow was installed by a peristaltic pump, set to a flow rate of 0.5 mL min^−1^. Once the measuring chamber was filled, measurements were started for 1 h. For acceptable frequency variations in phosphate buffer (typically below 30 Hz), the degradation experiment was started. Otherwise, another sensor was calibrated, coated, and assessed.

### Enzymatic degradation

The measuring chamber, the stirred container, and circulation tubes were kept filled with the phosphate buffer solution to avoid air bubbles. Enzyme solution was introduced into the stirred container and mixed for 1 min at 700 RPM. Afterwards, the peristaltic pump was switched on and set at a flow rate of 0.5 mL min^−1^. As soon as the enzyme solution circulated, measurements were started. Finally, once the enzymatic degradation was finished, measurements were kept for approximately one more hour.

## Results

The degradation of thin films made of PHB and PHO was investigated using scl-depolymerases from *P. lemoignei* LMG 2207 and recombinant mcl-depolymerases from *E. coli* DH5α harboring the plasmid pMAD8, respectively. To assess the degradation process, QCM measurements were carried out. The results revealed a sigmoidal degradation rate profile, consisting of an initial acceleration phase, an inflection point representing the highest degradation rate (*υ*_*0*_) and a subsequent deceleration phase (Supplementary Figure S2). The raw data were analyzed to obtain the degradation rate for each experiment and are compiled in Table 1.

**TABLE 1:** Degradation rates extracted from raw data processing according to the protein concentration for all degradation experiments.

	Protein concentration [μg mL^−1^]	Degradation rate *ʋ*_*0*_ [ng cm^−2^ s^−1^]
Degradation of PHB by scl-depolymerases	828.4	11.5
	414.2	17.2
	207.1	18.2
	145.0	14.4
	82.8	13.0
	41.4	7.8
	16.6	6.5
	4.1	2.9
	0.8	1.0
	0.2	0.2
Degradation of PHO by mcl-depolymerase	122.2	7.0
	24.4	8.7
	12.2	7.1
	2.4	2.3
	0.5	0.8
	0.1	0.2

**FIGURE 1:**
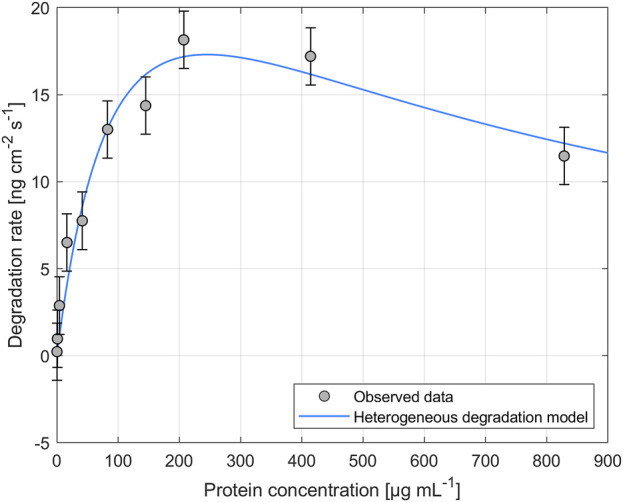
Degradation of PHB thin films exposed to depolymerases from *Pseudomonas lemoignei* LMG 2207 at various protein concentrations. The heterogeneous degradation model was fitted to the observed data through non-linear regression using the Levenberg-Marquardt algorithm. The degradation rate measurement for an enzyme concentration of *C*_*prot*_ = 82.8 µg mL^−1^ was carried out in triplicate and the standard deviation was used to calculate the error bars assuming a constant error.

**FIGURE 2:**
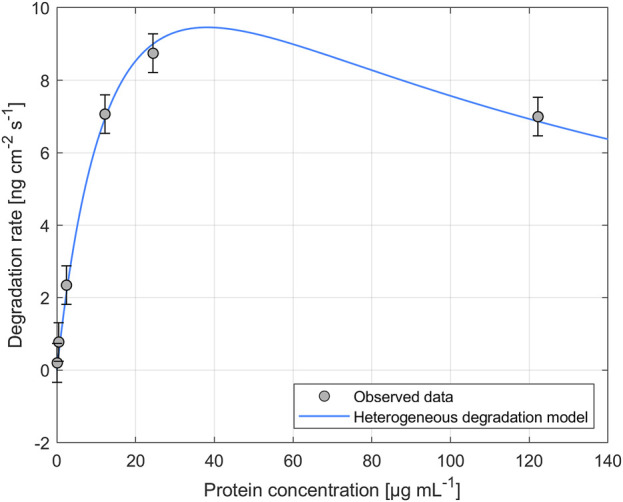
Degradation of PHO thin films exposed to recombinant mcl-depolymerases from *Escherichia coli* DH5α harboring the plasmid pMAD8, at various protein concentrations. The heterogeneous degradation model was fitted to the observed data through non-linear regression using the Levenberg-Marquardt algorithm. The degradation rate measurement for an enzyme concentration of *C*_*prot*_ = 2.4 µg mL^-1^ was carried out in triplicate and the standard deviation was used to calculate the error bars assuming a constant error.

The impact of different enzyme concentrations on degradation rate was investigated. The protein concentrations tested for PHB and PHO films differed, with a wider range of 0.2–828.4 µg mL^−1^ for PHB and a narrower range of 0.1–122.2 µg mL^−1^ for PHO. Consistent with findings in the literature for scl-PHA, QCM measurements revealed that the degradation rate initially increased with increasing enzyme concentration, reaching a maximum value, and followed by a decreasing degradation rate with further increasing enzyme concentrations. The enzyme concentration corresponding to the maximum degradation rate was higher when using scl-depolymerases to degrade PHB thin films than when using mcl-depolymerases to degrade PHO thin films. According to these observations, results were analyzed using the heterogeneous degradation model, which was fitted to the observed degradation rate data through non-linear regression with the Levenberg-Marquardt algorithm. Figures 1, 2 display the observed data and the corresponding fitted models for the degradation of PHB and PHO, respectively. We like to point out, that in the case of the used experimental set-up, the observed mass loss cannot directly be correlated with the hydrolysis rate of the enzymes. Although both enzyme classes are known to primarily produce water soluble monomers, dimers, and trimers as degradation products (Abe and Doi, 1999; Handrick et al., 2004; Martínez et al., 2015), the fragmentation of the PHA polymer into non soluble degradation products would lead to their deposition on the surface and would not be observed as a frequency change in the QCM measurement. However, due to the stability testing prior to each experiment in the absence of polymer degrading enzyme, mass loss as observed by a resonance frequency increase can be attributed to the degradation of the polymer by the enzyme into soluble products. We therefore refer to the constant *k'* of the heterogeneous degradation model as degradation rate constant, rather than hydrolysis rate constant as originally proposed by Mukai et al. By applying the kinetic model, it was determined that the maximum degradation rate for PHB films was 17.3 ng cm^−2^ s^−1^ and occurred at a scl-depolymerases concentration of 245.6 µg mL^−1^. For PHO films, the maximum degradation rate was 9.5 ng cm^−2^ s^−1^ and occurred at a mcl-depolymerases concentration of 38.2 µg mL^−1^.

The kinetic constants were obtained through non-linear regression analysis of QCM measurements and are compiled in Table 2. The adsorption equilibrium constant (*K*) represents the affinity of the enzyme to the substrate and reflects the initial adsorption step of the depolymerization process. The higher *K* value for mcl-depolymerases on PHO films compared to scl-depolymerases on PHB films indicates a stronger affinity of the mcl-depolymerases for the PHO substrate. Concerning the degradation rate constant (*k'*), it reflects the enzymatic degradation activity of the enzyme and represents the rate of mass reduction after adsorption. The lower *k'* value for mcl-depolymerases compared to scl-depolymerases suggests that mcl-depolymerases have a lower enzymatic efficiency in degrading PHO films. These results indicate that the enzyme-substrate interactions and degradation activity play key functions in the degradation of PHB and PHO films by scl- and mcl-depolymerases.

**TABLE 2:** Kinetic constants calculated by non-linear regression of experimentally observed data to the heterogeneous degradation model for the PHB and PHO degradation.

	Adsorption equilibrium constant (*K*) [mL µg^−1^]	Degradation rate constant (*k'*) [ng cm^−2^ s^−1^]
Degradation of PHB by scl-depolymerases	0.004	69
Degradation of PHO by mcl-depolymerase	0.026	38

## Discussion

The interaction strength between an enzyme and its substrate is represented by the constant *K* in the heterogeneous degradation model. The QCM measurements showed that the *K* value was six times higher for mcl-depolymerases and PHO when compared to scl-depolymerases and PHB. It indicates a stronger adsorption of mcl-depolymerase to PHO than the adsorption of scl-depolymerases to PHB. We attribute the differences in *K* values between these two systems to the higher hydrophobicity of the PHO polymer. Although structurally different, with a C-terminal substrate binding domain for scl-depolymerases (Behrends et al., 1996) and an N-terminal substrate binding domain for mcl-depolymerases (Gangoiti et al., 2012), both enzymes have a hydrophobic site that promotes absorption to the substrate. However, the presence of longer aliphatic side chains of the PHO polymer reduces the competitive effect of solvent absorption at the polymer surface when compared to the more polar PHB surface where water molecules will absorb to a higher extend, leading to a lower number of absorbed enzyme molecules in the equilibrium state.

The degradation rate of a biopolyester under the catalytic effect of an enzyme is expressed by the constant *k'* in the heterogeneous degradation model. The QCM measurements showed that the *k'* value was approximately twice as high for scl-depolymerases and PHB than for mcl-depolymerases and PHO. Possible explanations, apart from the different specific environments of the Serine-Histidine-Aspartate motif (i.e., catalytic triad), can be found in the different nature of the PHA-backbones. PHA hydrolysis takes place at the ester forming carbonyl groups of the polymers. For mcl-PHAs these are sterically more challenging due to their longer aliphatic side chains. This sidechain length also reduces the quality of the leaving group and the nucleophilicity of the carbonyl group.

Considering the mechanism of the degradation of PHA as proposed by Mukai et al., this leads not only to a reduced maximum degradation rate for the mcl-depolymerase when compared to the scl-depolymerases of this study but also to a lower optimal enzyme concentration since the fast adsorption accompanied by a relatively slow and rate-limiting hydrolysis process leads to faster surface crowding by the absorbed enzyme molecules on the substrate surface (Figures 1, 2).

In conclusion, we believe that these findings will support the process of developing applications for mcl-PHAs and the critical consideration of end-of-life scenarios. A low abundance of natural mcl-PHA producers in the environment, concomitant scarcity of mcl-PHA depolymerases and the observed lower degradation rate constant when compared to scl-PHA depolymerases, invite to consider new routes for monomer reutilization and upcycling. In this respect, enzymatic engineering might offer a viable solution to improve the enzymatic degradation of PHA depolymerases by addressing their substrate binding and hydrolysis profiles.

## Data Availability

The raw data supporting the conclusion of this article will be made available by the authors, without undue reservation.
