# Supplementary material for: Degradation kinetics of medium chain length Polyhydroxyalkanoate degrading enzyme: a quartz crystal microbalance study
Source: Front Bioeng Biotechnol. 2023 Dec 14;11:1303267. doi: 10.3389/fbioe.2023.1303267 (PMC10756687; doi:10.3389/fbioe.2023.1303267)
Supplement: Supplementary file 1 [file DataSheet1.docx]

Supplementary Material

Degradation kinetics of medium chain length polyhydroxyalkanoate degrading enzyme: a quartz crystal microbalance study

Fabien Millan, Nils Hanik*

*** Correspondence:** Nils Hanik: nils.hanik@hes-so.ch

# Supplementary Figures and Tables

## Supplementary Figures


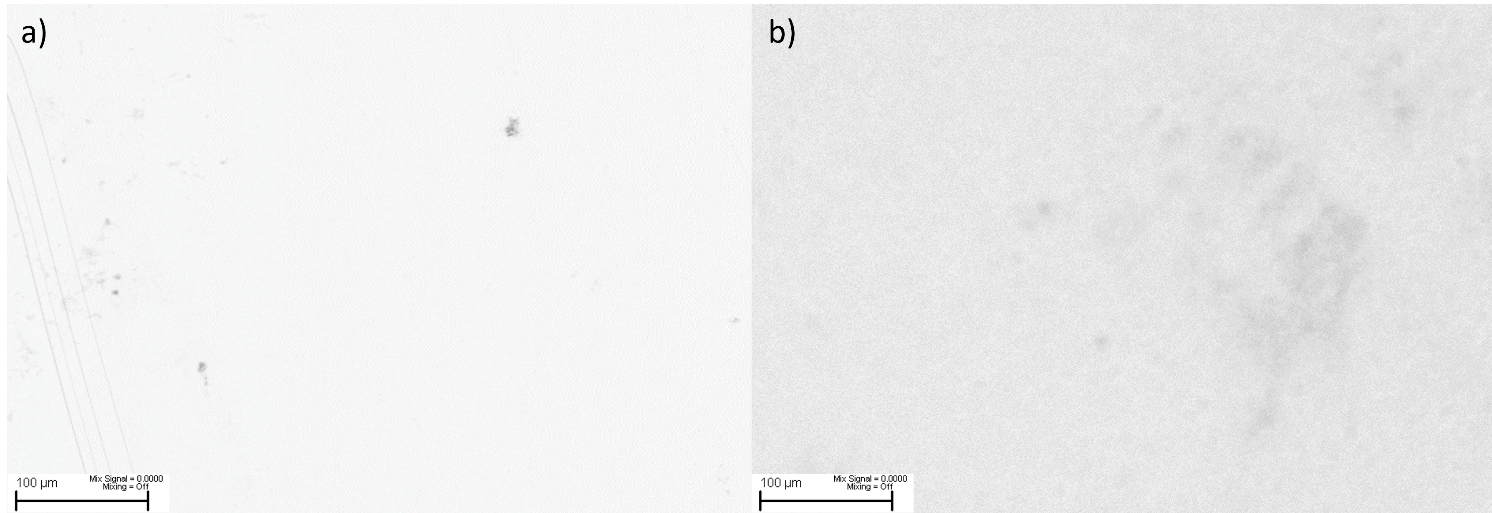


Supplementary Figure 1: SEM images (X-Max, Oxford instruments) of the quartz crystal before (a) and after coating (b) with polhydroxybutyrate. The polymeric sample was sputtered with gold to promote conductivity. The accelerating voltage was set to 8.00 kV, working distance between 14 to 16 mm.


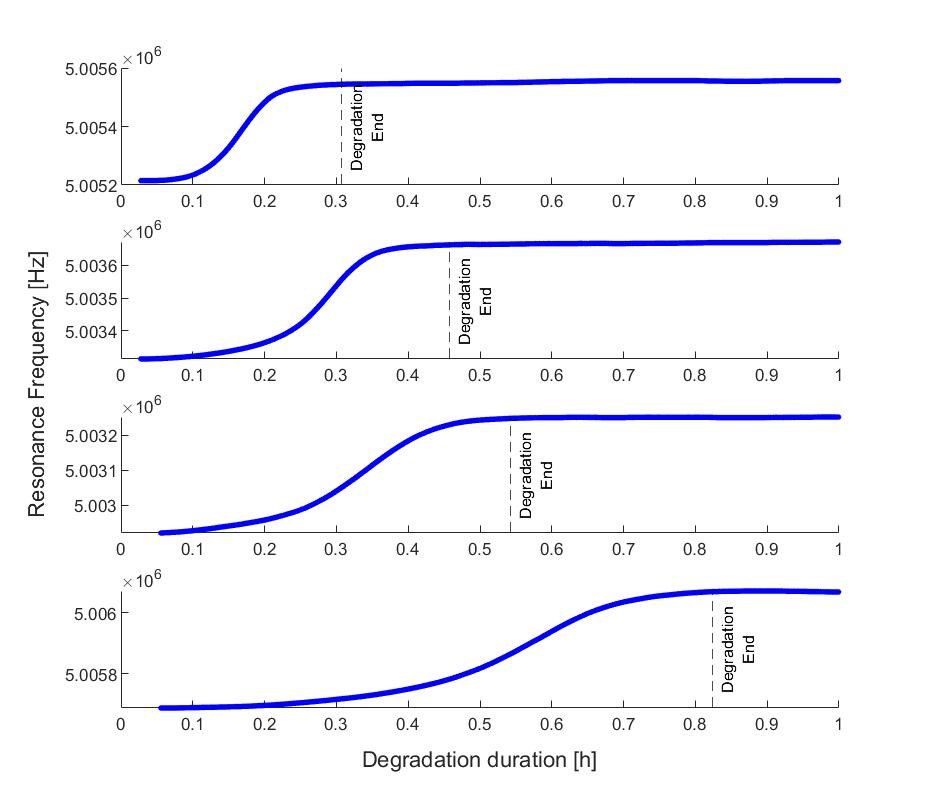


**Supplementary Figure 2.** Evolution of the RF over time degradation and the identified degradation end of the degradation experiment A (*Cprot* = 414.21 µg mL^-1^ and dilution 1:6), B (*Cprot* = 82.84 µg mL^-1^ and dilution 1:30), C (*Cprot* = 41.42 µg mL^-1^ and dilution 1:60) and D (*Cprot* = 16.57 µg mL^-1^ and dilution 1:150).
